# The relationship between trunk control, spinal posture, and spinal mobility in patients with multiple sclerosis: a cross-sectional study

**DOI:** 10.55730/1300-0144.5778

**Published:** 2023-12-25

**Authors:** Taşkın ÖZKAN, Nezehat Özgül ÜNLÜER, Mustafa Ertuğrul YAŞA, Buse KORKMAZ, Gönül VURAL

**Affiliations:** 1 Department of Therapy and Rehabilitation, Vocational School of Health Services, Giresun University, Giresun, Turkiye; 2 Department of Physiotherapy and Rehabilitation, Gülhane Faculty of Physiotherapy and Rehabilitation, University of Health Sciences, Ankara, Turkiye; 3 Department of Neurology, Faculty of Medicine, Ankara Yıldırım Beyazıt University, Ankara, Turkiye

**Keywords:** Multiple sclerosis, postural balance, posture, spine

## Abstract

**Background/aim:**

Trunk control, which plays a key role in balance and mobility, decreases in patients with multiple sclerosis (PwMS) and many parameters such as sensory, motor, and musculoskeletal systems affect trunk control. The aim of this study was to compare trunk control, spinal mobility, and spinal posture in PwMS with healthy controls and investigate the relationship between trunk control with spinal posture and spinal mobility in PwMS.

**Materials and methods:**

The study was completed with 38 PwMS and 38 healthy controls with matched age and sex. Trunk control was evaluated with the Trunk Impairment Scale (TIS). Spinal posture and mobility were evaluated in sagittal and frontal planes using an IDIAG M360 Spinal Mouse. Spinal posture was evaluated in upright, maximum flexion, extension, left and right lateral flexion positions, and spinal mobility was evaluated from upright to flexion, extension, right and left flexion positions in sagittal and frontal planes.

**Results:**

TIS scores, thoracic mobility angles (from upright to flexion and left lateral flexion), lumbar mobility angles (from upright to extension and right lateral flexion) and lumbar posture angle (maximum right lateral flexion) were lower, and thoracic posture angles (upright and maximum extension) were higher in PwMS than healthy controls (p < 0.05). No significant difference was found between other spinal postures and mobility values. In addition, there was only a negative relationship between thoracic spinal mobility from upright to extension and trunk control in PwMS (r = −0.349; p = 0.032).

**Conclusion:**

These findings indicate the importance of early detection of trunk disturbances in PwMS. Thus, even in the early stages of multiple sclerosis, detailed trunk assessment will guide the implementation of comprehensive exercise programs.

## 1. Introduction

Multiple sclerosis (MS), an idiopathic autoimmune-inflammatory disease of the central nervous system (CNS), is characterized pathologically by demyelination in the brain, spinal cord, and visual nerves and following axonal degeneration [1]. The progression of MS is incredibly unpredictable and varied. The majority of patients experience episodes of reversible neurological deficits at the beginning of the illness, which are followed by progressive neurological decline over time. The severity of disability caused by MS increases especially with axonal loss [2]. MS symptoms are often associated with the most extensively myelinated areas of the central nervous system, although they are notoriously variable. Three common symptoms, fatigue, muscle weakness, and psychological difficulties, are associated with impaired quality of life in patients with MS (PwMS) [3].

There is a close relationship between posture and psychological state. Body posture can be used as a component of bodily expression, especially in people who cannot reflect their emotions with facial expressions [4]. Canales et al. [5] defined depression as a multidimensional disease that affects the body as a whole and they reported a relationship between the recurrence of depressive symptoms and postural misalignment. Bae et al. [6] stated that progressive muscle fatigue with activity and secondary pain may be factors that cause changes in spinal alignment. Additionally, it is also known that upright posture reduces fatigue and improves psychological symptoms in depressed patients [7]. Considering the high rates in PwMS of depression with a lifetime prevalence of ~50% and fatigue with a lifetime prevalence as high as 80%, postural alterations can also be expected in this patient group [8,9].

Muscle function alterations are common symptoms of MS. They include stiffness or involuntary muscle movements, as well as weakness that reduces the functional capacity of the muscle [10]. Trunk muscles also suffer from the progressive weakness and stiffness caused by MS [11]. However, trunk control is a prerequisite not only for achieving sitting and standing but also for different extremity movements. During various functional tasks, it is very important that the trunk is displaced without loss of balance, but the stiffness of the trunk muscles can cause a decrease in trunk mobility in PwMS [12]. Impaired trunk control and accompanying falls were identified in PwMS [13,14]. Therefore, it is important to investigate the factors that contribute to the reduction of trunk control in MS in a cause-effect cycle.

Lanzetta et al. [15] examined some features of postural control during functional activities performed in a sitting position and they showed that trunk control decreased in PwMS when compared to healthy controls. They suggested further investigations should be made to clarify other important contributors to impaired trunk balance. Ebrahimi Meymand et al. [16] described some spinal postural abnormalities in PwMS compared to healthy individuals with similar characteristics. However, to the best of our knowledge, the relationship between spinal posture, spinal mobility, and trunk control has not been investigated yet. Accordingly, this study was planned to investigate some of the physical characteristics of the trunk including spinal posture, spinal mobility, and trunk control in comparison with healthy controls and to determine the relationships between spinal posture, spinal mobility, and trunk control in PwMS. The hypothesis of this study is that trunk control, spinal posture, and spinal mobility will be lower in PwMS compared to healthy controls. Another hypothesis is that weaker trunk control will be related to abnormalities of spinal posture and spinal mobility.

## 2. Materials and methods

### 2.1. Study design

This research was designed as a cross-sectional study and was carried out at Health Sciences University, Faculty of Physical Therapy and Rehabilitation, from February 2023 to April 2023. Written consent was obtained after informing the participants in this research about the procedure. All the procedures were conducted in accordance with the Declaration of Helsinki. Ethics committee approval was obtained with decision number 16 at the meeting of Ankara Yıldırım Beyazıt University Health Sciences Ethics Committee on 27.10.2022.

### 2.2. Participants

In the context of our research, 40 patients diagnosed with MS by a neurologist and 40 healthy controls of similar age and body mass index were included in the study. Healthy controls were recruited as a control group from a local community center through poster advertising. The inclusion criteria for PwMS were (1) being between the ages of 18 to 65, (2) being diagnosed with MS disease by a specialist neurologist, (3) having a score above 24 according to Mini-Mental State Examination (MMSE), (4) not having an MS attack in the last 3 months, (5) not being included in the physiotherapy and rehabilitation program in the last 3 months, and (6) not having an additional neurological disease or circulatory system or vision problem that may cause balance disorder. The exclusion criteria for PwMS were (1) using corticosteroids in the past 4 weeks, (2) changing medications used in the last 1 month, (3) having congenital spinal deformities (e.g., scoliosis, spinal dysraphism, vertebral anomaly), and spinal disc herniation (symptomatic individuals), and (4) being pregnant. The inclusion criteria for healthy controls were (1) being between the ages of 18–65, and (2) having a score above 24 according to MMSE. The exclusion criteria for healthy controls were (1) having spinal deformities (e.g., scoliosis, spinal dysraphism, vertebral anomaly), (2) spinal disc herniation, and (3) severe biomechanical limitations of the hip, knee, and ankle.

### 2.3. Procedure

Demographic characteristics of all cases and clinical characteristics of PwMS (duration of illness, MS type, and disability level) were recorded. Disability level was evaluated by a neurologist with the Expanded Disability Status Scale (EDSS). Outcome measurements were performed in the same sequence by the same physiotherapist in a peaceful, well-lit room. All evaluations were completed in approximately 25 min. Between the measurements, 2-min rest periods were given.

### 2.4. Outcome measures

Trunk control was evaluated with the Trunk Impairment Scale (TIS). It evaluates stability while seated, selective lateral flexion, and selective rotation of the lower and upper parts of the trunk in order to determine static sitting balance, dynamic sitting balance, and trunk coordination. The TIS consists of 17 items. The maximum results for the subscales measuring trunk coordination, sitting balance, and dynamic sitting balance are 7, 10, and 6 points, respectively. A higher TIS score (which ranges from 0 to 23) indicates better trunk control [17]. TIS is a reliable and valid measure of trunk control for PwMS [18].

**Figure 1:**
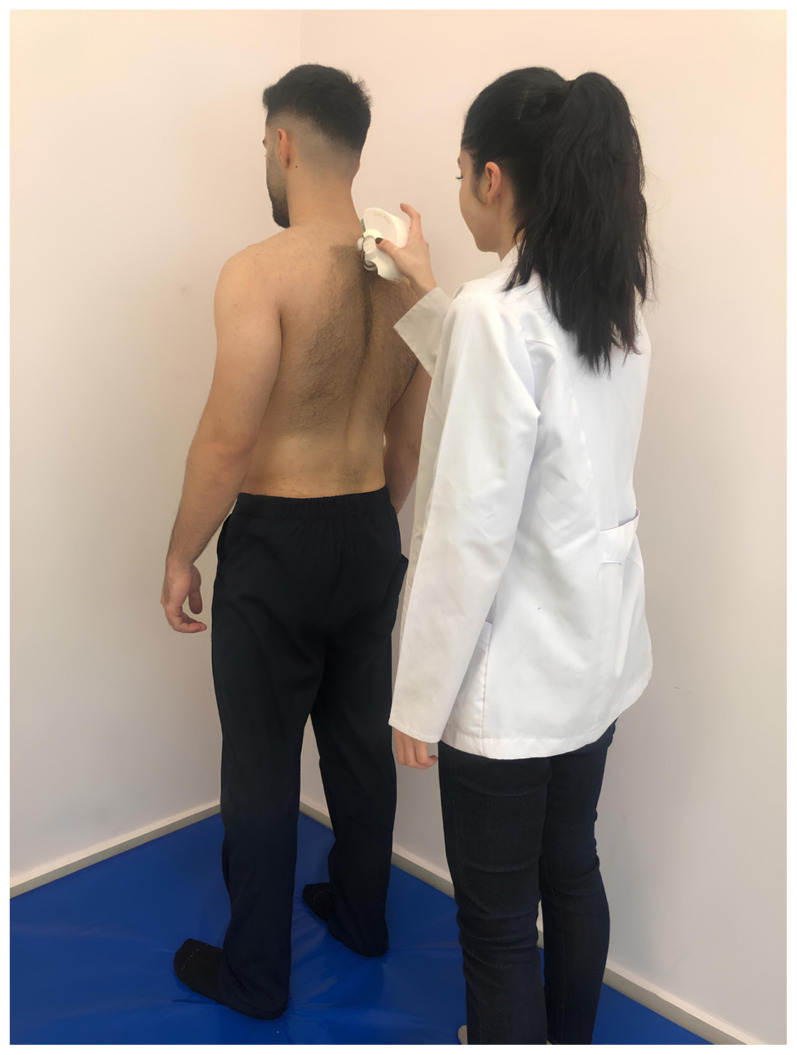
Upright position.

**Figure 2:**
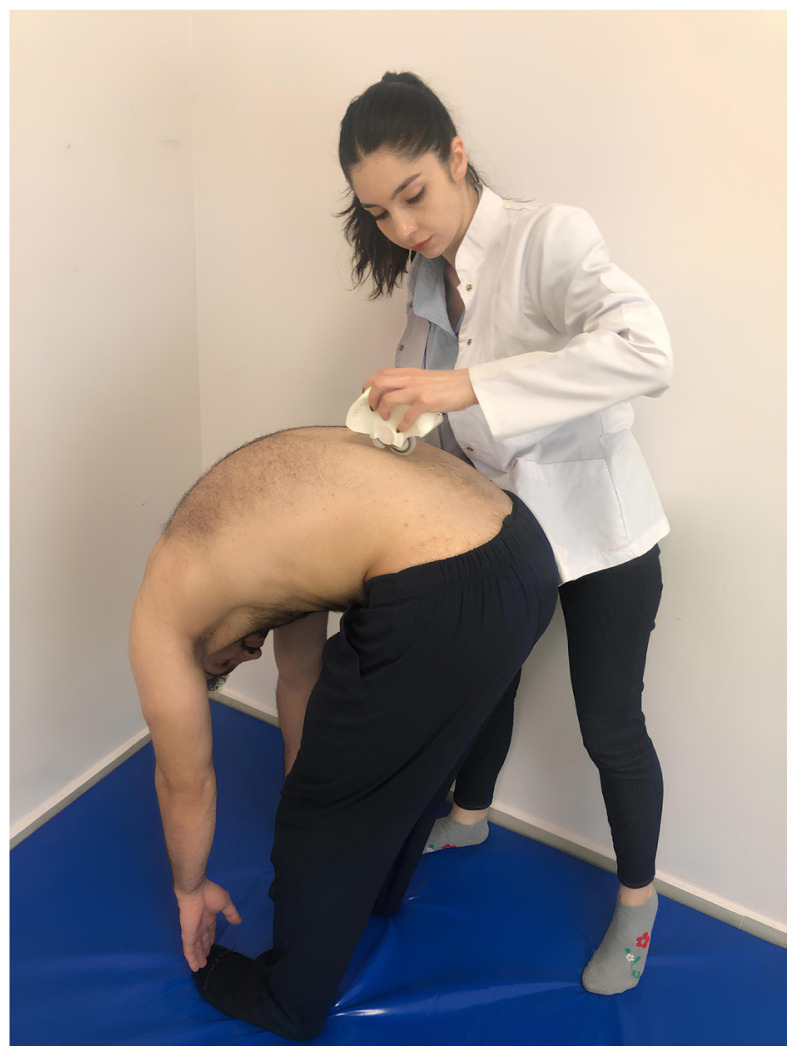
Maximum flexion position.

**Figure 3:**
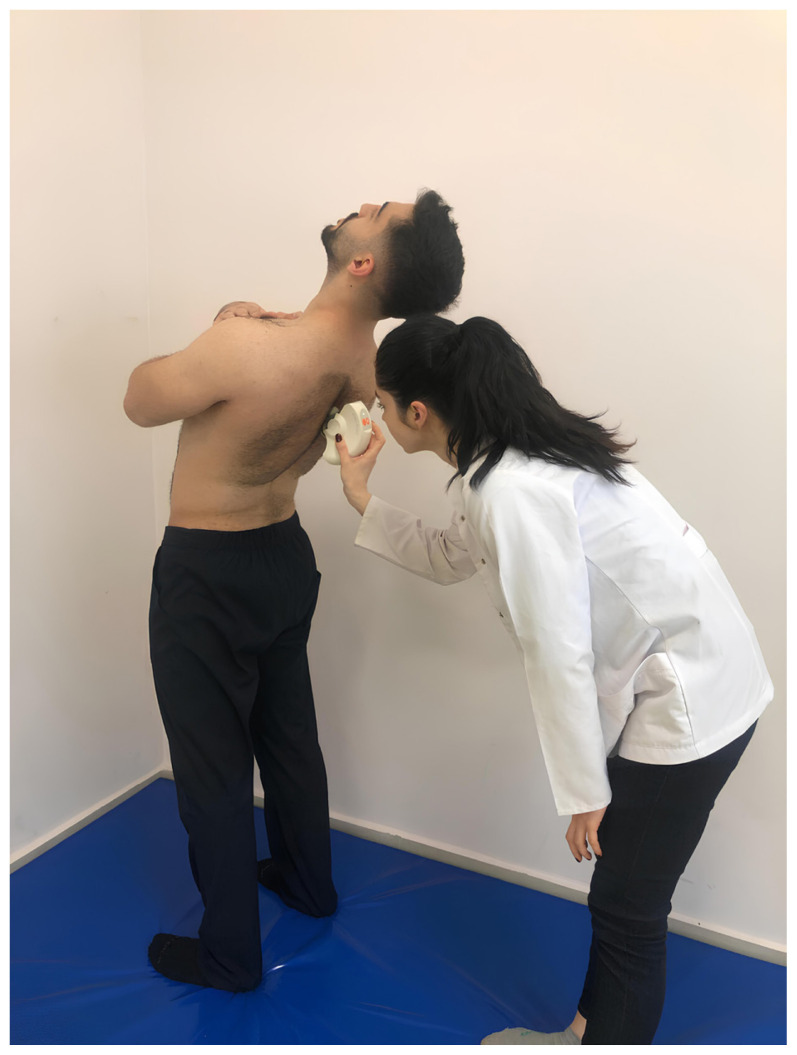
Maximum extension position.

**Figure 4:**
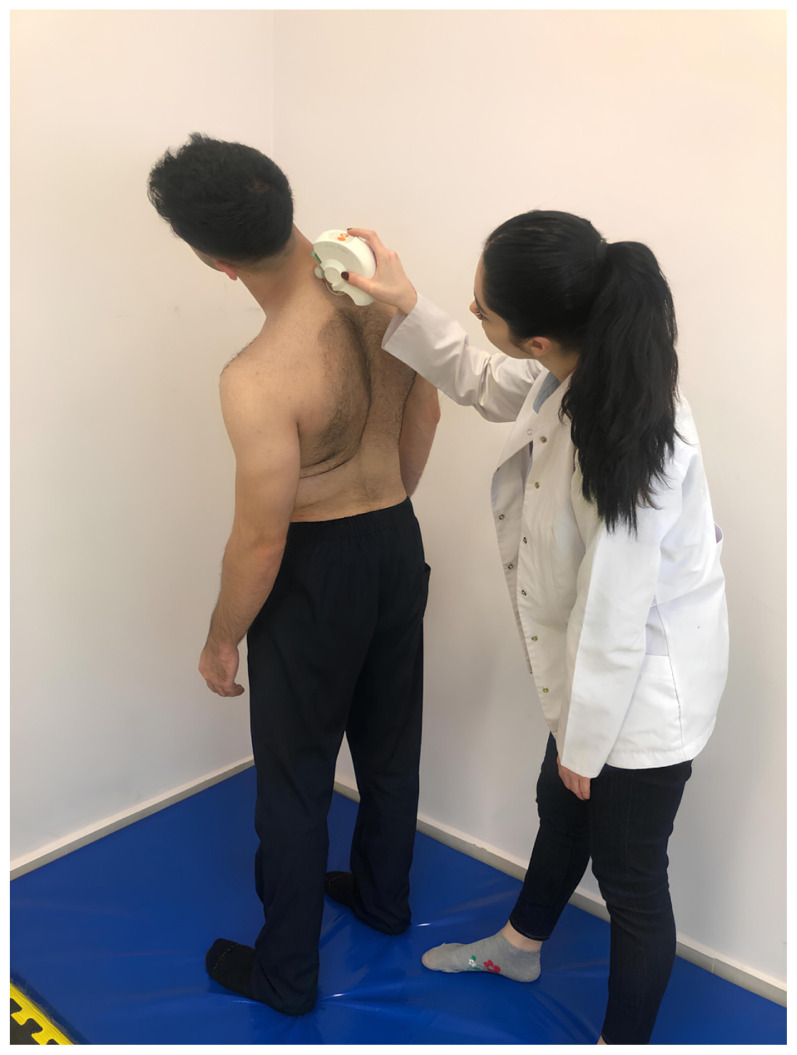
Maximum left lateral flexion position.

**Figure 5:**
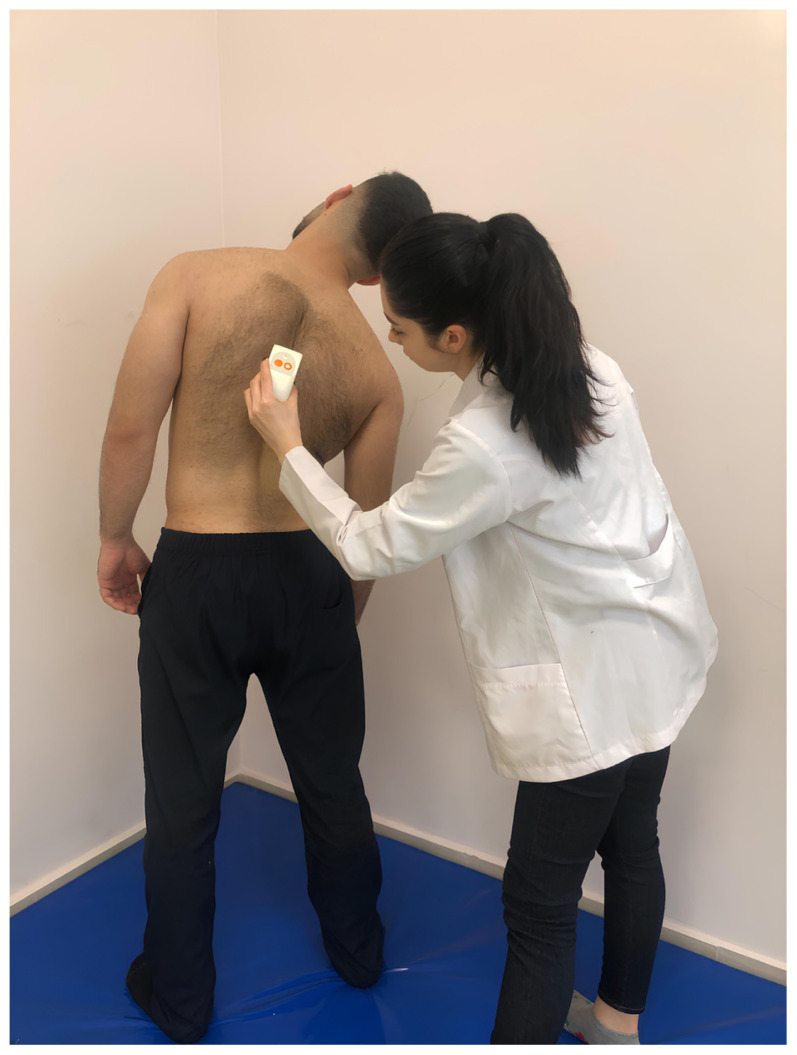
Maximum right lateral flexion position.

Spinal posture and mobility were evaluated using an IDIAG M360 Spinal Mouse (IDIAG, Fehraltorf, Switzerland). The Spinal Mouse is a noninvasive, computer-assisted electromagnetic measurement instrument that gives information about the curve and movement of the spine. This information enables analysis of segmental posture and movement of the spine in the sagittal and frontal planes. A computer receives data from the Spinal Mouse wirelessly and concurrently at 1.3 mm intervals and 150 Hz. The subjects were told to stand up straight with their feet shoulder-width apart and their weight equally distributed between the two lower extremities for assessment of spinal posture while they were naked from the waist up. In this position, spinous processes were palpated and marked with a cosmetic pen. The Spinal Mouse was moved with the person in five stances (neutral stance, maximum flexion, maximum extension, maximum right lateral flexion, and maximum left lateral flexion) by rotating both wheels at a constant speed and applying pressure to the spinous processes between the 7th cervical vertebra and the 3rd sacral vertebra. For the sagittal and frontal planes, respectively, positive values represent anterior posture (kyphosis) or movement (flexion) and right-side posture, whereas negative values represent posterior posture (lordosis) or movement (extension) and left-side posture. Spinal posture and spinal mobility measurements were performed once after one trial, in a total of five positions (Figures 1–5) [19,20].

### 2.5. Statistical analysis

The post hoc power analysis for the research was calculated using the G*Power software package (G*Power, Version 3.0.10, Franz Faul, Universität Kiel, German) [21]. The effect size (d) of the study was calculated as 1.37 as a result of the calculation carried out using TIS total data in which the total number of samples was 76, and the power of the study (1- β) was calculated as 0.99 with a 5% margin of error (a = 0.05). For statistical analyses, SPSS 22.0 (SPSS Inc., Chicago, USA) was used. The Statistical Package for Social Sciences (SPSS) Version 20 statistical program was used for statistical evaluation of the data. Data normality was tested using the Shapiro-Wilk test. Mean and standard deviation are used to represent normally distributed data, and median (IQR25-75) is used to represent nonnormal distributed data. Gender variables are expressed as frequency and percentage (%). The chi-square test was used to assess the difference between the groups in terms of sex. Intergroup differences were analyzed with the Independent Samples T-Test and Mann-Whitney U Test. The association between the variables in the PwMS group was determined using Spearman and Pearson correlation analysis. Statistical significance was set at alpha <0.05. The findings of the correlation analysis were divided into five categories: 0.81–1.00 (very good correlation), 0.61–0.80 (good correlation), 0.41–0.60 (moderate correlation), 0.21–0.40 (fair correlation), and 0.00–0.20 (poor correlation).

## 3. Results

The inclusion criteria were not met by 2 out of 40 participants. The MMSE scores for 2 PwMS were below 24 points. The study was completed with 38 PwMS. Of these, 33 of 38 patients were Relapsing-Remitting type and 5 of them were Secondary Progressive MS. Of the controls, 2 of the 40 healthy controls that were evaluated for the study did not match the criteria for inclusion and the study was completed with 38 healthy controls.

Participant demographic and disease characteristics are shown in Table 1. For demographic factors including age, BMI, andsex, there was no difference between the groups (p > 0.05, Table 1).

Static sitting, dynamic sitting, coordination subtests, and TIS total scores were lower in PwMS than in healthy individuals, and this difference was found to be statistically significant (p < 0.05, Table 2).

When the spinal posture values were compared, thoracic posture angles in the sagittal plane in the upright and maximum extension positions were higher, and lumbar posture angle in the frontal plane in maximum right lateral flexion position was lower in PwMS than in healthy controls, and these differences were found to be statistically significant (p < 0.05, Table 2). There was no significant difference between the groups for all other spinal posture parameters (p > 0.05, Table 2).

When the spinal mobility values were compared, thoracic mobility angles in movement from upright to flexion (sagittal plane) and left lateral flexion (frontal plane) and lumbar mobility angles in movement from upright to extension (sagittal plane) and right lateral flexion (frontal plane) were lower in PwMS than in healthy controls, and these differences were found to be statistically significant (p < 0.05, Table 2). There was no significant difference between the groups for all other spinal mobility parameters (p > 0.05, Table 2).

When examining the association between trunk control with spinal posture and mobility, except for the thoracic angle from upright standing to extension in the sagittal plane, there was no significant relationship between the results in PwMS (Table 3).

## 4. Discussion

Although trunk control has been investigated in PwMS in the literature, to our knowledge, the relationships between spinal posture, spinal mobility, and trunk control have not been adequately studied. We compared spinal posture, spinal mobility, and trunk control between PwMS and healthy controls of similar age, and also assessed the relationship between spinal posture, spinal mobility, and trunk control. Consistent with our hypotheses, trunk control, thoracic mobility angles (from upright to flexion and left lateral flexion), lumbar mobility angles (from upright to extension and right lateral flexion), and lumbar posture angle (maximum right lateral flexion) were lower and thoracic posture angles (upright and maximum extension) was higher in PwMS than healthy controls. In addition, there was a negative relationship between thoracic spinal mobility from upright to extension and trunk control in PwMS.

Trunk control is essential for movement, balance, and gait [22]. Since trunk control is the key point for movement, in the literature it was shown to be very important not only for lower extremity mobility and instability but also for upper extremity functional skills in PwMS [23]. Studies reported that forward-looking postural adjustments of the trunk while standing, dynamic trunk stability while sitting, and endurance in the trunk muscle system are impaired in PwMS [14,24]. In our study, consistent with the literature, trunk control, including static sitting, dynamic sitting, and trunk coordination, decreased in PwMS. Ozen et. al. compared trunk control between PwMS with an EDSS score of 5.5 points and healthy controls [25]. In their study, they found trunk control, which they evaluated with the core stability test, was weaker in PwMS subjects than in healthy controls. Raats et al. [26] reported that trunk impairments were detected throughout the full range of disability levels in PwMS, including mild disability. In addition, in the literature, there are studies investigating the relationship between trunk control, balance, functional mobility, and falls in neurologic diseases [22,24,27,28]. Ünlüer et al. [22] demonstrated that trunk motor control was related to balance, functional mobility, and gait capacity in PwMS with mild to moderate disability. Normann et al. [24] investigated the relationship between trunk control with balance and walking in PwMS with mild to moderate disability and found moderate relationships between dynamic trunk control while sitting with walking distance and walking speed. Interestingly, although the relationship of trunk control with lower extremity mobility, upper extremity functional activities, and respiratory functions were investigated [22–25], studies investigating the relationship of trunk control with spinal mobility and posture were not noticed. So, to contribute to the literature, this is the first study that investigates the relationship between trunk control, spinal posture, and spinal mobility in PwMS. Our results show that there was no association between trunk control with spinal posture and mobility, except for the thoracic angle from upright standing to extension in the sagittal plane. This relationship between trunk control and spinal mobility is also a low-level relationship. The results of the current study did not reveal a significant relationship between trunk control with spinal posture and mobility. This situation led to the consideration that trunk control may be affected by different neuromuscular parameters other than spinal posture and mobility in mild to moderate PwMS. Additionally, this relationship may exist in a population where spinal posture and mobility parameters worsen in the advanced stages of PwMS. At the same time, we think that investigating this relationship is very valuable because evaluating the thoracic, lumbar, and sacral vertebrae separately from the early period is important for the treatment of the problematic sections of the spine.

Spinal posture is affected by many neuromuscular mechanisms, as well as structural disorders, familial factors, mental status, lifestyle, and daily habits [29,30]. In our study, thoracic kyphosis was significantly higher in an upright posture (in the sagittal plane), and lumbar lordosis values were noticeably higher, although not significantly, in PwMS compared to healthy controls. Ebrahimi Meymand et al. [16] reported that the lumbar lordosis degree in women with mild PwMS was significantly higher than in healthy controls. Muhtaroğlu et al. [31] showed that the thoracic kyphosis degree in PwMS was significantly higher than in healthy individuals. In this study, the thoracic kyphosis angle was 46.13 ± 55.64 in the PwMS, similar to our results found for a thoracic sagittal degree in upright posture. Although studies about spinal posture in PwMS are limited, the literature appears to be compatible with the results of our study. When the posture of the spine in maximum flexion position in the sagittal plane was examined, PwMS could flex their thoracic, lumbar, and sacral regions, similar to healthy controls. In the maximum extension position, while they were able to extend the lumbar and sacral regions sufficiently, it was observed that they did not and/or could not provide sufficient extension in the thoracic region. This suggests that there may be a structural or functional joint limitation in the thoracic region, or that patients may develop this in order to compensate for the loss of muscle strength, muscle imbalance, loss of proprioception, or balance disorder that may exist in the trunk. When the posture of the spine was examined in the frontal plane in the maximum right and left lateral flexion position, a decrease in the lumbar angle during right lateral flexion movement was observed in PwMS compared to healthy controls. Although there was no significant difference between them, it appears that the lumbar angle during left lateral flexion movement is less in PwMS compared to the healthy controls. If the median values are examined, lateral flexion angles were between −15° and +13° in PwMS, and between −19° and +20° in healthy controls. PwMS had approximately 11° less lateral flexion movement in the frontal plane than healthy controls. These results suggest joint limitation in the lumbar region or compensation secondary to MS complications, as stated before for the thoracic region. The fact that the majority of patients in this study had mild levels of disability may indicate that postural changes have not yet progressed to a very significant level but worsened compared to healthy individuals; that is, progressive postural deterioration may be experienced.

When spinal mobility was examined in the sagittal plane, the thoracic flexion degree during flexion from an upright stance and the lumbar extension degree during extension from an upright stance were significantly lower in PwMS. Additionally, the thoracic degree was lower during left lateral flexion from upright posture in the frontal plane and the lumbar degree was lower during right lateral flexion. These results indicate a remarkable loss of mobility in the thoracic and lumbar spine of PwMS. This suggests that the angular decrease in spinal posture may also affect spinal mobility. These results illustrate the need to evaluate spinal mobility as well as spinal posture from an early stage, and the importance of including applications aiming to improve spinal posture and increase spinal mobility in treatment protocols.

When the current literature is examined, to the best of our knowledge, there is no other study examining spinal posture and mobility in PwMS, apart from the studies by Ebrahimi Meymand et al. [16] and Muhtaroğlu et al. [31]. In this context, our study is the first to examine spinal posture and mobility in detail. Although it is a different neurological disease, Yaşa et al. [32] examined spinal posture and mobility in Parkinson’s disease. They determined that thoracic kyphosis increased, and lumbar lordosis decreased but no significant difference was found. In addition, they stated that thoracic and lumbar mobility were significantly lower than in healthy controls. Although the results for lumbar lordosis are compatible with our study, the results for thoracic kyphosis are different from ours. We think that the most important factor affecting this result is the different neurological disease groups.

The study has limitations as results refer only to the early phase of PwMS. Further studies are needed to examine the relationship between the results of advanced imaging methods for lesions in the central nervous system and trunk control, spinal posture, and spinal mobility in PwMS.

In conclusion, the results of this study demonstrated that trunk control, thoracic and lumbar mobility decreased and thoracic and lumbar posture altered in PwMS than healthy controls, and the present study did not reveal an important relationship between trunk control and spinal posture and mobility. These results suggest that practices aimed at correcting spinal posture, increasing spinal mobility, and trunk control should be included in rehabilitation approaches in PwMS with mild to moderate disability.

## Figures and Tables

**Table 1:** Demographic and clinical characteristics of PwMS and healthy controls.

Characteristics	MS group (X ± SD)	Control group (X ± SD)	p
Age, years	41 ± 11	42 ± 11	0.856
BMI, kg/m^2^	24.68 ± 4.08	24.77 ± 3.76	0.920
Gender (female/male), n (%)	28 (73.7)/10 (26.3)	26 (68.4)/12 (31.6)	0.613
EDSS, score	2.51 ± 0.93	-	-
Duration of illness, years	8.4 ± 5.4	-	-

MS: Multiple Sclerosis, BMI: Body Mass Index, EDSS: Expanded Disability Status Scale, X: Mean, SD: Standard deviation,

* p < 0.05.

**Table 2:** Comparison of trunk control, spinal posture, and spinal mobility test results of PwMS and healthy controls.

				MS group median (IQR25/75)	Control group median (IQR25/75)	p
**Trunk control**						
Trunk Impairment Scale (point)		Static sitting		7 (7/7)	7 (7/7)	**0.013** *
		Dynamic sitting		6 (6/10)	10 (10/10)	**<0.001** *
		Coordination		6 (4/6)	6 (6/6)	**<0.001** *
		Total		19 (17/23)	23 (23/23)	**<0.001** *
**Spinal posture**						
Sagittal (degree)	Upright posture		Thoracic	49 (41/56)	41(32/46)	**<0.001** *
			Lumbar	−29 (−34/−36)	−19 (−28/−12)	0.077
			Sacral	10(2/18)	9 (0/15)	0.767
	Maximum flexion		Thoracic	56 (50/61)	52 (46/57)	0.383
			Lumbar	24 (18/31)	28 (21/35)	0.212
			Sacral	60 (49/72)	61 (56/68)	0.448
	Maximum extension		Thoracic	44 (33/46)	31 (26/37)	**0.001** *
			Lumbar	−28 (−35/−15)	−30 (−42/−10)	0.702
			Sacral	−9 (−18/−4)	−9 (−25/12)	0.421
Frontal (degree)	Upright posture		Thoracic	2 (−1/6)	4(1/7)	0.098
			Lumbar	−1(−4/1)	−3(−7/0)	0.082
			Sacral	4(1/6)	4 (1/7)	0.971
	Maximum left lateral flexion		Thoracic	−7 (−12/−5)	−10 (−14/−7)	0.066
			Lumbar	−15 (−20/−9)	−19 (−26/−13)	0.089
			Sacral	−3 (−7/1)	−5 (−8/−1)	0.205
	Maximum right lateral flexion		Thoracic	21 (16/27)	21 (18/26)	0.442
			Lumbar	13 (10/18)	20 (11/23)	**0.020** *
			Sacral	13 (9/16)	11 (7/15)	0.149
**Spinal mobility**						
Sagittal (degree)	From upright to flexion		Thoracic	8 (0/13)	14 (8/20)	**0.005** *
			Lumbar	56 (42/59)	46 (36/59)	0.323
			Sacral	51 (39/60)	51 (42/65)	0.497
	From upright to extension		Thoracic	−7 (−14/−3)	− −9(−15/1)	0.963
			Lumbar	−1 (−6/7)	−6(−16/4)	**0.021** *
			Sacral	−18 (−25/−13)	−16 (−24/−5)	0.158
Frontal (degree)	From upright to left lateral flexion		Thoracic	−12 (−16/−7)	−15 (−19/−10)	**0.009** *
			Lumbar	−13 (−21/−8)	−14 (−22/−8)	0.496
			Sacral	–7 (−10/−4)	−8 (−11/−4)	0.246
	From upright to right lateral flexion		Thoracic	17 (14/25)	17 (13/22)	0.759
			Lumbar	15 (10/19)	22 (13/27)	**0.001** *
			Sacral	10 (6/14)	7 (5/10)	0.141

MS: Multiple Sclerosis, IQR: Interquartile range, TIS: Trunk Impairment Scale,

* p < 0.05.

**Table 3:** The relationship between trunk control and spinal posture and mobility in PwMS.

			Trunk Control	
			r	p
**Spinal posture**				
Sagittal (degree)	Upright posture	Thoracic	0.061	0.716
		Lumbar	−0.138	0.408
		Sacral	−0.006	0.970
	Maximum flexion	Thoracic	0.054	0.747
		Lumbar	−0.107	0.523
		Sacral	0.083	0.619
	Maximum extension	Thoracic	−0.221	0.183
		Lumbar	0.006	0.971
		Sacral	−0.209	0.209
Frontal (degree)	Upright posture	Thoracic	0.036	0.829
		Lumbar	0.013	0.936
		Sacral	0.035	0.836
	Maximum left lateral flexion	Thoracic	0.127	0.446
		Lumbar	−0.022	0.896
		Sacral	−0.022	0.989
	Maximum right lateral flexion	Thoracic	0.032	0.849
		Lumbar	−0.134	0.421
		Sacral	0.058	0.731
**Spinal mobility**				
Sagittal (degree)	From upright to flexion	Thoracic	0.010	0.951
		Lumbar	0.017	0.918
		Sacral	0.082	0.624
	From upright to extension	Thoracic	−0.349	**0.032** *
		Lumbar	0.147	0.378
		Sacral	−0.251	0.128
Frontal (degree)	From upright to left lateral flexion	Thoracic	0.054	0.750
		Lumbar	−0.032	0.847
		Sacral	−0.105	0.529
	From upright to right lateral flexion	Thoracic	0.058	0.731
		Lumbar	−0.059	0.723
		Sacral	−0.007	0.968

MS: Multiple Sclerosis,

* p < 0.05.
